# Effect of acupuncture on hormone level in patients with gastrointestinal dysfunction after general anesthesia: A study protocol for a randomized controlled trial

**DOI:** 10.1097/MD.0000000000019610

**Published:** 2020-04-03

**Authors:** Lisha Liu, Xiuli Yuan, Lei Yang, Jingyuan Zhang, Jing Luo, Guangqiang Huang, Jian Huo

**Affiliations:** a Mianyang Affiliated Hospital, Chengdu University of Traditional Chinese Medicine, Mianyang; b ChengDu University of Traditional Chinese Medicine, Chengdu; c Mianyang Third Hospital, Mianyang; d Mianyang Anzhou Hospital, Mianyang, China.

**Keywords:** acupuncture, general anesthesia, laparoscopy, postoperative gastrointestinal dysfunction, RCT, study protocol

## Abstract

**Background:**

Postoperative gastrointestinal dysfunction (PGD) refers to one of the common postoperative complications. Acupuncture can facilitate the recovery of PGD, whereas no therapeutic schedule of acupuncture has been internationally recognized for treating PGD. In the present study, a scientific trial protocol has been proposed to verify the feasibility of acupuncture in treating gastrointestinal dysfunction after laparoscopic cholecystectomy under general anesthesia. We conduct this protocol to investigate whether acupuncture recovery gastrointestinal dysfunction by influencing the expression of gastrointestinal hormone.

**Method:**

The present study refers to a randomized, evaluator blinded, controlled, multi-center clinical trial; it was designed complying with the Consolidated Standards of Reporting Trials (CONSORT 2010) as well as the Standard for Reporting Interventions in Controlled Trials of Acupuncture (STRICTA). The subjects will be taken from the inpatients having undergone laparoscopic surgery of Mianyang Affiliated Hospital of Chengdu University of traditional Chinese medicine, Mianyang Third Hospital and Mianyang Anzhou Hospital. Based on the random number yielded using SPSS 25.0 software, the qualified subjects will be randomly classified to the experimental group and the control group. Therapies will be performed 30 min once after operation, the experimental group will be treated with acupuncture, while the control group will receive intravenous injection of granisetron. The major outcome will be the time to first flatus, and the secondary outcomes will include the time to first defecation, abdominal pain, dosage of analgesia pump, abdominal distention, nausea, vomiting, gastrointestinal hormone, as well as mental state. The efficacy and safety of acupuncture will be also assessed following the principle of Good Clinical Practice (GCP).

**Discuss:**

A standardized and scientific clinical trial is conducted to assess the efficacy and safety of acupuncture for gastrointestinal dysfunction after laparoscopic cholecystectomy under general anesthesia. The aim is to objectively evidence and improves the clinical practice of acupoint prescription, as an attempt to promote the clinical application of this technology.

## 1 Introduction

Postoperative gastrointestinal dysfunction (PGD) refers to a common postoperative complication, capable of causing many clinical symptoms (e.g., nausea, vomiting, abdominal pain, bloating, constipation, as well as diarrhea).^[1]^ PGD is likely to cause intestinal paralysis, acute gastric dilatation, and obstructions in severe cases, thereby affecting patients’ quality of life and prolonging the hospital stay of the patient.^[2]^ The specific mechanism of PGD remains unclear. It has been known that PGD is tightly associated with general anesthesia, gastrointestinal hormones, electrolyte disturbance, as well as abdominal inflammation.^[3]^ For the routine treatment of PGD, fasting, enema, oxygen inhalation, analgesia, fluid supplement, acid–base regulation, infection prevention, as well as gastrointestinal decompression are covered.^[4]^ Granisetron, domperidone, cisapride, and everypan refer to the typical drugs for PGD. Domperidone and cisapride are likely to induce adverse reactions of cardiovascular system; granisetron can cause fever and headache, and side effects of nausea and vomiting may be attributed to evermopan.^[5]^ As reported in the mentioned literature, the side effects and potential risks of these drugs limit their wide application.

Over the past few years, Enhanced recovery after surgery (ERAS)^[6]^ (i.e., applying all types of evidence-based therapies in the perioperative period to mitigate surgical complications, expedite postoperative recovery and shorten hospital stay of the patients) has aroused increasing attention by surgeons; acupuncture has been considered a part of ERAS by some researchers and has been progressively applied in treating postoperative complications.^[7,8]^ In 1998, the National Institutes of Health issued a consensus statement that acupuncture can act as a feasible treatment for postoperative nausea and vomiting.^[9]^ Acupuncture refers to an alternative therapy of traditional Chinese medicine (TCM), numerous experimental and clinical studies reported that acupuncture has a significant two-way regulatory effect on the movement, secretion and absorption of the digestive system.^[10,11]^ By stimulating nerves, especially vagus nerve, acupuncture can achieve the regulating effect on gastrointestinal function.^[12]^ Lu^[13]^ reported that electroacupuncture at *Zusanli* (ST36) regulated gastric motility in rats by vagus and sympathetic reflex, mediated by M2/3 and β1/2 receptors, respectively; in this procedure, vagus reflex played a major role. As suggested in a clinical research,^[14]^ acupuncture at *Zusanli* (ST36) can stimulate the peroneal nerve, and subsequently the pulse signal was transmitted to the vagus nerve via the sciatic nerve, release the 1-amino acid decarboxylase to elevate the neurotransmitter level. As revealed from an experimental research,^[15]^ electroacupuncture at *Neiguan* (PC6) can stimulate the sensory fibers of the median nerve and subsequently stimulate the solitary tract nucleus of the vagus nerve, so the neurotransmitters were release. As the research regarding gastrointestinal diseases has been leaping forward, a growing number of scientists believe that gut and brain are closely related, and the brain-gut axis links central nervous system and gastrointestinal tract.^[16]^ Signals from the brain are capable of affecting gastrointestinal motility, whereas information transmitted from the gastrointestinal tract can affect brain function as well.^[17]^ Brain-gut peptide refers to a type of small molecular peptide with double distribution in gastrointestinal tract and brain, being critical to gastrointestinal motility.^[18]^ Peripheral ghrelin has been suggested to be vital to regulate gastrointestinal function, while ghrelin in central nervous system can considerably affect gastrointestinal activity.^[19]^An experiment also suggested that electroacupuncture can reduce auxin and neuropeptide Y in rats and reduce their food intake.^[20]^ Excessive release of 5-hydroxytryptamine (5-HT) and substance P (SP) from gastrointestinal tract might cause intestinal dysfunction, while scalp acupuncture can down-regulate the expression level of 5-HT and SP in irritable patients to facilitate the recovery of gastrointestinal function.^[21]^

Some systematic reviews have also verified the feasibility of acupuncture for PGD and have drawn a positive conclusion.^[22,23]^ For the defects of research quality, statistics and research methods, the clinical results should be interpreted rigorously, and their scientific basis remain insufficient.^[24]^ Scalp acupuncture is a part of acupuncture therapy to treat diseases by stimulating specific location of the head area, showing a considerable effect on numerous diseases resulting from the brain.^[25]^ The acupuncture prescriptions for PGD primarily involve the points located in trunk and limbs.^[26]^ In the present clinical trial, the acupoints in the head area will be chosen in accordance with brain-gut axis theory, which is a novel exploration and attempt to apply acupuncture for PGD. By referencing to the design of similar studies,^[27]^ 18 patients having undergone laparoscopic surgery were pre-tested strictly following the evaluation and treatment process of this study. These subjects were randomly classified to “experimental group” (acupuncture) and “control group” (granisetron); the experimental group (16.31 ± 2.21 h) had a shorter average time to first flatus than the control group (26.14 ± 3.53 h). According to the preliminary results, acupuncture can effectively relieve gastrointestinal dysfunction after laparoscopic cholecystectomy.

## 2 Methods

### 2.1 Trial design

**Figure 1:**
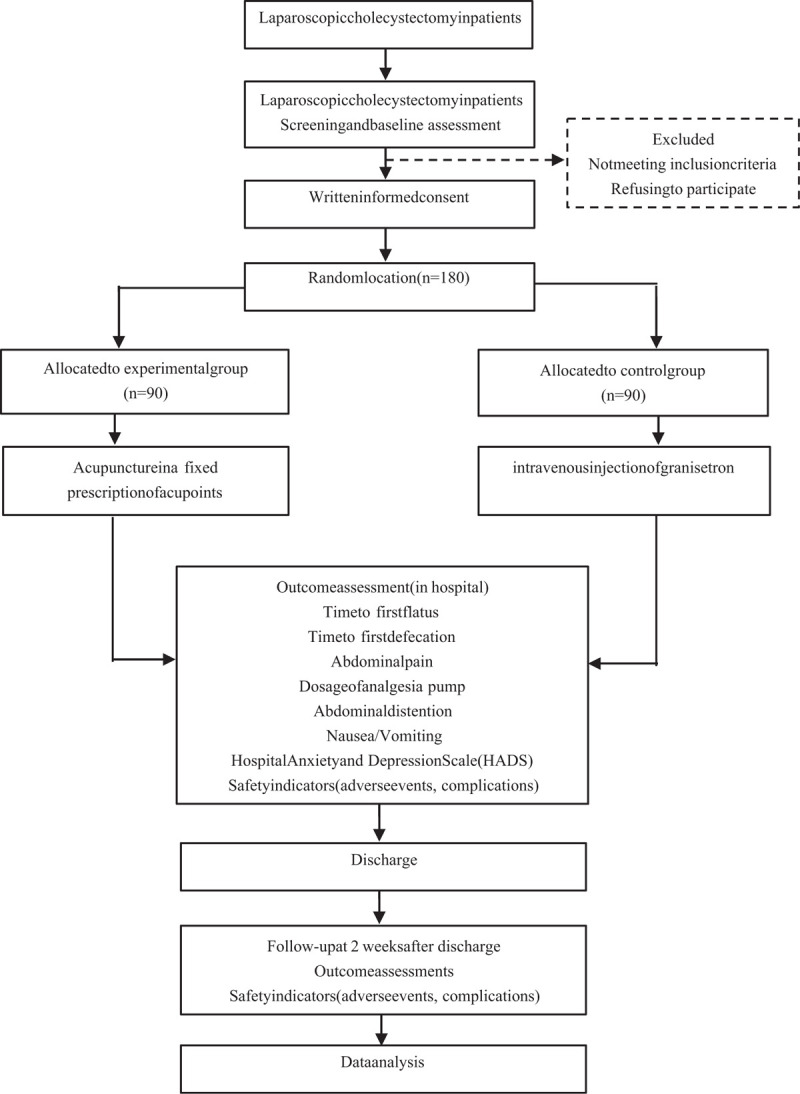
Participant flow diagram.

**Figure 2:**
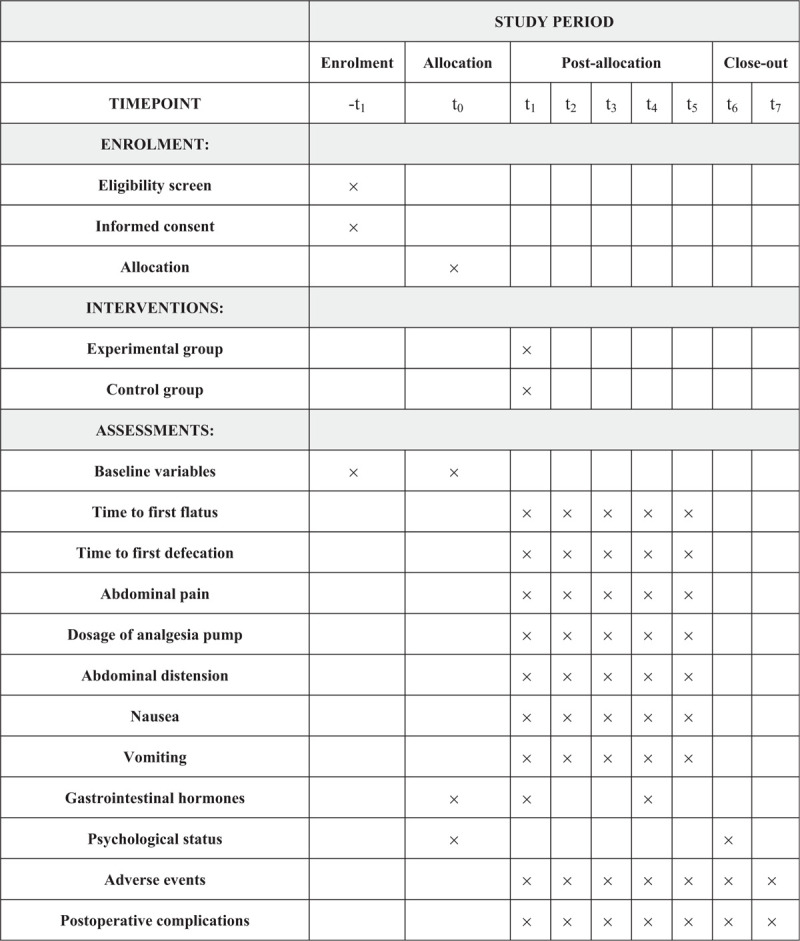
SPIRIT schedule of schedule of enrollment, interventions, and assessments. t1 is the day before surgery, t0 is the day of the surgery, t1 is 0 to 2 h after surgery, t2 is 2 to 6 h after surgery, t3 is 6 to 12 h after surgery, t4 is 12 to 24 h after surgery, t5 is 24 to 48 h after surgery, t6 is the day of the discharged, t7 is 2 weeks after patients discharged from the hospital.

This study refers to a randomized, evaluator blinded, controlled, multi-center clinical trial to be conducted in Mianyang Affiliated Hospital of Chengdu University of TCM, Mianyang Third Hospital, Mianyang Anzhou Hospital, China. Eligible participants will be randomly classified into the experimental group (n = 90) and the control group (n = 90) at a ratio of 1:1. The patients will receive one of the following interventions 30 min after laparoscopic cholecystectomy, which are acupuncture and intravenous injection of granisetron. The results will abide by the Consolidated Standards of Reporting Trials (CONSORT)^[28]^ guidelines as well as Standards for Reporting Interventions in Clinical Trials for Acupuncture (STRICTA)^[29]^ and Standard Protocol Items: Recommendations for Interventional Trials (SPIRIT) guidelines.^[30]^ This study (protocol version 2.0, May 25, 2018) was registered at the Chinese Clinical Trials Registry (ChiCTR-1800016991). Figure 1 illustrates the research design and Figure 2 presents the trial schedule.

### 2.2 Participants

All subjects will be diagnosed by two surgical experts and one anesthesiologist in accordance with the inclusion and exclusion criteria. The potential participants meeting all the inclusion criteria and match none of the exclusion criteria should communicate with the research assistant about the study face to face. The research assistant will conduct a series of disease assessment and safety assessment to eligible patients interested in the study. After signing the informed consent, subjects will be randomly split into two groups and received different treatment.

### 2.3 Recruitment

During the study, three research assistants should go to the general surgery department of the research centers three times a week to screen out subjects. Eligible patients will be identified and invited to the study. The research assistants will provide written informed consent to the patient and elucidate the details of the study (e.g., objective, scope, procedure, potential benefits, and risks). The patient will sign the informed consent in the presence of the research assistants who can also accept oral informed consent if the participants are unable to read.

### 2.4 Inclusion criteria

Subjects will be included if meeting all the following criteria of the study:

1. the age between 18 and 70 years;
2. the operation type is laparoscopic cholecystectomy, and the anesthesia type is general anesthesia, complying with the I to II classification standard of American Society of anesthesiologists;
3. the intravenous analgesia pump will be employed postoperatively;
4. the operation time is 2 ± 1. 0 h;
5. having not undergone any other clinical trials during this study;
6. without any cognitive impairment, aphasia, mental disorder, and other communication barriers;
7. having participated in the trial and sign the informed consent voluntarily.

### 2.5 Exclusion criteria

Subjects will be excluded if meeting any of the following criteria:

1. obesity (body mass > 80 kg), during pregnancy and lactation, with a fertility or pregnancy plan within recent 3 months;
2. with severe cardiovascular disease, central nervous system disease, diabetes mellitus, as well as psychosis;
3. administrated with gastric motility drugs within 24 h before operation or gastric tube keeping after operation;
4. with severe gastrointestinal disease or previous history of PGD;
5. averse to acupuncture treatment, or have aphasia, audio-visual disorders;
6. with intraoperative blood loss ≥1000 mL or hemoglobin <70 g/L;
7. with the history of drug abuse.

### 2.6 Elimination standard

Subjects will be eliminated from the trial if meeting any of the following conditions:

1. laparoscopy must be converted to laparotomy;
2. refusal to continuous treatment for various reasons;
3. with severe syndromes (e.g., biliary fistula and peritonitis) during the treatment;
4. transferred to other specialist treatments;
5. having become comatose or die;
6. cannot participate in the follow-up for various reasons;
7. researchers accidentally included the subjects not meeting the inclusion criteria;
8. less compliance with the treatment regulations and failed to provide information that might be critical to the evaluation.

### 2.7 Randomization, allocation concealment

SPSS 25.0 will be employed to generate the random number. A specially appointed researcher will be responsible for random grouping to reduce selection bias and eliminate confounding factors. Subjects will be randomly classified into the experimental group, and the control group in the proportion of 1:1, each group will cover 90 participants. The recruiters will obtain the sequence number from the researcher responsible for assigning when eligible subjects get recruited in the trial. To further reduce the bias and prevent communication, subjects from the two groups will receive treatment in separate rooms.

To prevent researchers’ prejudice from confusing the results, the sealed envelope method will be employed to blind randomization. The distribution sequence number of subjects will be placed in the sealed and opaque envelope, so the recruiters and evaluators cannot know the allocations. To ensure strict confidentiality, the envelope will be not transparent even under strong light. To avoid confusion, the subject's name and birthdates will be written on the outside of the envelope with carbon paper. The recording of the information regarding the sealed envelope and the subject's details will be made by a video, and a copy of the outer label of the sealed letter will be attached to the group distribution card in the envelope. Afterwards, the video will be checked by another researcher to ensure that the envelopes are sealed. The whole process of opening the envelopes will be also recorded by the video as well.

### 2.8 Blinding

Due to the characteristics of acupuncture clinical trials and the high popularity of acupuncture in China, it is unlikely to thoroughly blind subjects in this trial, nor to make acupuncturists blind in treatment allocation. As a result, acupuncturists will not be involved in the assessment, and different groups will undergo treatment in different rooms to ensure that it is impossible for participants to impact the group allocation. The evaluators and statisticians will conduct their assigned work independently and be unaware of the group allocation throughout the whole study.

### 2.9 Components of the ERAS protocol

All participants in the two groups will receive perioperative care in accordance with a standardized ERAS protocol. A systematic review of colonic surgery recommended 17 vital elements of the ERAS protocol,^[31]^ components of our ERAS protocol met 14 of it (Table 1). For experimental group, acupuncture will act as additional components of ERAS.

**Table 1:**
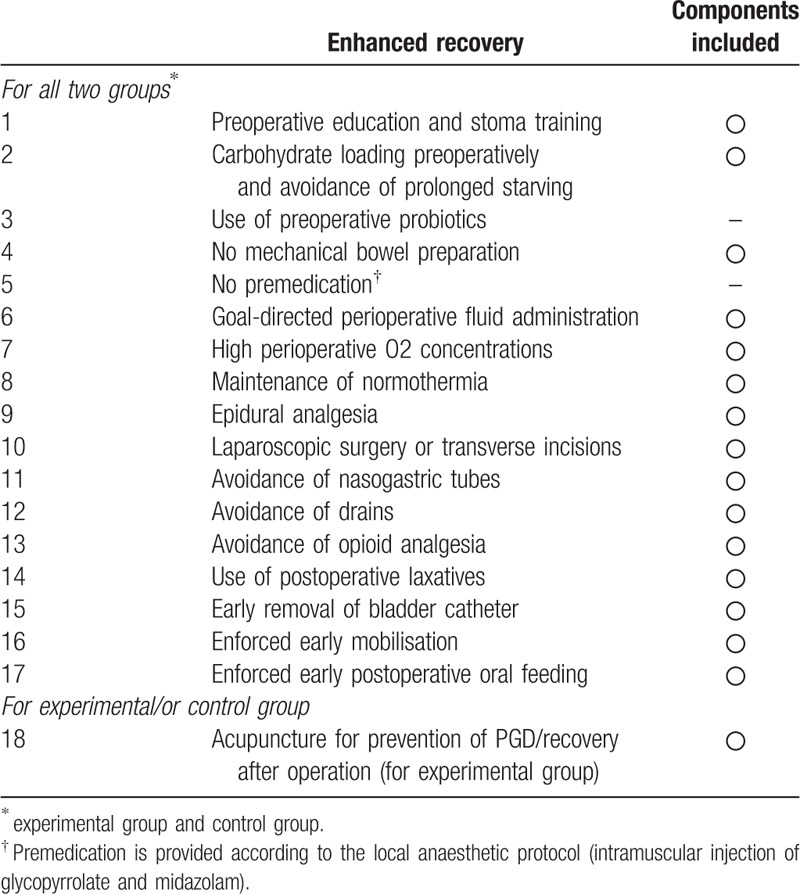
Components of an enhanced recovery program.

### 2.10 Intervention

**Figure 3:**
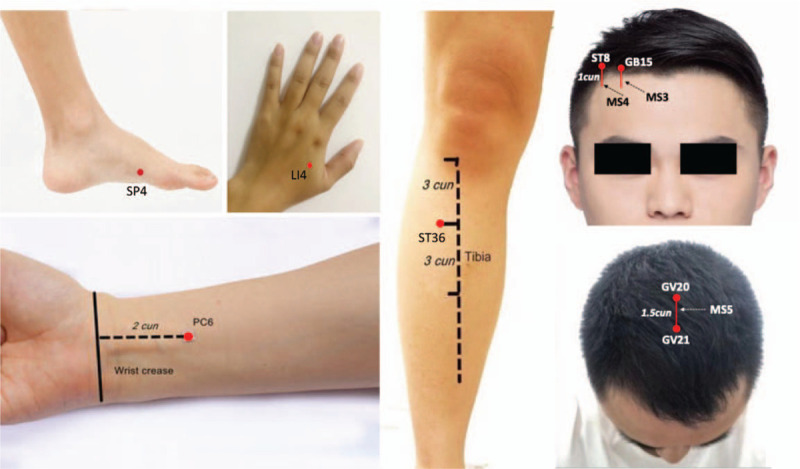
The acupoints location of the fixed acupuncture prescription.

The treatment strategy of this study will be collectively formulated by experienced acupuncturists, surgeons and anesthesiologists. After laparoscopy, all the subjects will be fasted and in supine position without pillow, the intervention is going to be conducted once 30 min after surgery. The experimental group will receive the treatment of sterile disposable stainless-steel needles (*Huatuo* medical instruments Co. Ltd., Suzhou, China; 0.3 mm × 40 mm/0.3 mm × 25 mm). Acupoints will be located abiding by the World Health Organization Standard Acupuncture Point Locations in the Western Pacific Region.^[32]^ The fixed prescription includes *Hegu* (LI4), *Neiguan* (PC6), *Zusanli* (ST36), *Gongsun* (SP4), lateral line 2 of forehead (MS3), lateral line 3 of forehead (MS4), and middle line of vertex (MS5) (Fig. 3), all acupoints will be bilaterally selected except for middle line of vertex (MS5). The acupuncture needles will be inserted into acupoints after the routine sterilization; subsequently, it will be evenly lifted, inserted and then twisted to *Deqi* after reaching the standard depth; the operation of each acupoint will take 30 s. The needles will be maintained on the trunk and limbs for 30 min and on the head for 6 h after *Deqi*. Acupuncture will be performed by two acupuncturists registered in China and with over 3 years of clinical experience. They will receive the relevant training before the experiment, the training courses covered acupoint positioning, acupuncture operation skills and communication skills. The control group will be intervened by professional surgical nurses, the treatment will be intravenous injection of granisetron with 3 mg/3 mL (drug approval No. H20030161, Sichuan Taiji Pharmaceutical Co., Ltd).

### 2.11 Outcome indicators

#### 2.11.1 Baseline data

The following baseline data will be recorded by the established personal information questionnaire, namely, demographic characteristics (e.g., gender, age, height, and weight), general clinical conditions (e.g., operation time, bleeding volume, anesthesia time, and intraoperative infusion volume), as well as basic vital signs (e.g., heart rate, blood oxygen, blood pressure, and respiration).

#### 2.11.2 Primary outcome

Time to first flatus: the researcher will inform the subjects and their families to help record the interval between the time of subjects returning to the ward after the operation to the time of first flatus after operation.

#### 2.11.3 Secondary outcome

**Figure 4:**
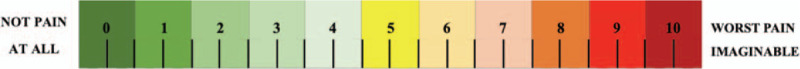
Visual analog scale.

1. Time to first defecation: Researchers will inform the subjects and their families to assist in recording the interval between the time of subjects returning to the ward after the operation to the time of first defecation after operation.
2. Abdominal pain: The Visual Analog Scale (VAS, Fig. 4) will be adopted to assess the degree of postoperative abdominal pain of subjects,^[27]^ which is to be recorded by researchers at t_1_ to t_5_.
3. Dosage of analgesia pump: Considering that the use of analgesia pump will affect the degree of abdominal pain,^[33]^ therefore, the dosage of analgesia pump used by participants after operation will be recorded by researchers at t_1_ to t_5_.
4. Abdominal distention: Likert scale (see Table 2) will be adopted to assess the degree of abdominal distention^[30,34]^ and recorded by researchers at t_1_ to t_5_.
5. Nausea: The VAS scale will be employed to assess the degree of nausea^[27]^ and recorded by researchers at t_1_ to t_5_.
6. Vomiting: The subjects and their families will help record the times of vomiting at t_1_ to t_5_.
7. Gastrointestinal hormones: Blood samples will be taken 8 h before operation, 30 min after operation, and 24 h after operation to detect the level of gastrointestinal hormones. Enzyme-linked immunosorbent assay will be used to detect the levels of serum gastrin, motilin, ghrelin, 5-HT in the two groups. Three milliliters of blood will be harvested from the vein in the fasting state. The blood samples will be kept at ambient temperature for 30 min and then centrifuged at 3500 rpm for 10 min; the serum will be stored in a refrigerator at −70°C for testing. Serum samples will be collected by clinical centers, and relevant tests will be performed by Chengdu Lilai Biotechnology Co., Ltd.
8. Psychological status: The Hospital Anxiety and Depression Scale (HADS) will be adopted to assess the psychological status of the subjects at t_0_ and t_6_.^[35]^ HADS has two subscales of anxiety and depression. There will be seven problems for anxiety and depression respectively. The anxiety and depression are expressed by the score of 0 to 7 points (no symptoms), 8 to 10 points (suspicious symptoms), 11 to 21 points (obvious symptoms). The first evaluation will be conducted 8 h before the operation, and the second one will be conducted on the day of discharge.

**Table 2:**
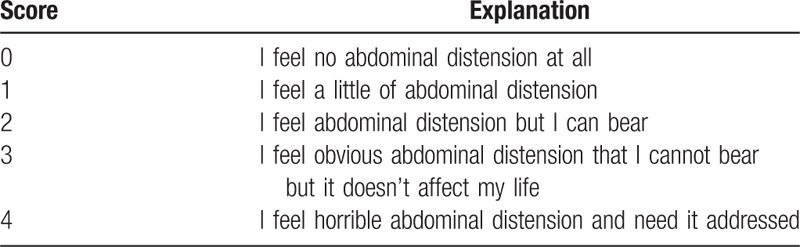
Presentation of the Likert-type scale.

### 2.12 Adverse events and complications

The participants of this study should voluntarily report information regarding adverse events to researchers (e.g., pallor, fainting during acupuncture treatment, sweating, dizziness, local hematoma, bleeding, unbearable prickling, as well as continuous severe pain more than 1 h after acupuncture). The adverse events regarding the acupuncture (e.g., sticking of the needle, broken needles, and bent needles) will be reported by acupuncturists. All adverse events should be completely registered on the adverse events page of case report forms (CRFs). Researchers are supposed to ascertain the occurrence of adverse events while recording all details (e.g., date, time, extent, and correlation with treatment). Data on postoperative complications will be recorded during hospitalization and within 2 weeks after discharge, any complications that will threaten the life of the subjects, or serious adverse events will be also recorded. The researchers will seek emergency medical assistance and report the serious adverse events immediately.

### 2.13 Data collecting and monitoring

The result data of this study will be recorded by a special evaluator on the paper CRF. To more effectively conduct the observation of indicators and data collection, the CRF contained all information (e.g., observation time, observation indicators, adverse event records, as well as safety assessment). The result evaluator should fill in the relevant information quickly and accurately in accordance with the principle of CRF. The evidence-based medicine center of Chengdu University of TCM will be responsible for data monitoring every 3 months. The acupuncturists and statisticians cannot access or obtain the data during the whole assessing process.

### 2.14 Quality control

To ensure the quality of the study, methodology experts, acupuncturists, surgeons, and anesthesiologists discussed and revised the protocol repeatedly. Before the launching of the project, all researchers must receive unified training covering research scheme, test process, operation specification, etc. The quality controller directly appointed by the project leader will check the CRF every 2 weeks. The original data on the CRF cannot be modified. If it needs to be modified, the reason for the modification shall be stated, and the name of the modifier shall be written next to the modification. The conference will be held by researchers every 3 months to report and discuss the process of the study.

### 2.15 Sample size and statistical analysis

#### 2.15.1 Sample size calculation

The total sample size of the study will be calculated by the formula based on a two-group, parallel, controlled design: n = (*Z*_1−*α*/2_ + *Z*_1−*β*/2_)^2^ × (*σ*2 1 + *σ2 2*)/*δ*^2^, where n denotes the sample size of each group, *σ* represents the standard deviation, and δ indicates the clinical significance between groups. At an α = 0.05, power = 80%, and given a bilateral test, *Z* scores of *Z*_1−*α*/2_ = 1.96 and *Z*_1−*β*/2_ = 0.84 will be obtained from the *Z* score table. Eighteen subjects recruited from February 2018 to March 2018 will be recruited in the pilot trial, and the primary outcome measure will be the time to first flatus: S1 = 2.21 and S2 = 3.53. Based on preliminary trial and clinical experience, the reduction of 4 h in the time to first flatus will be defined as our clinical expectation. Accordingly, the rounded sample size in each group is n ≈78. Assuming that the dropout rate is 15%, 180 subjects (90 in each group) will be needed in the two groups.

#### 2.15.2 Statistical analysis

1. Analysis procedures sample distribution: The number of dropouts and the rate will be described and analyzed for each data set, and the reasons for any dropouts are elucidated.
2. Balance comparison: The subjects’ general data will be compared to assess the comparability of the two groups.
3. Efficacy analysis: The subjects’ time to first flatus will be used as the primary outcome measure to assess the efficacy and safety of acupuncture in treating PGD. The means (χ) and standard (SD) deviations will be calculated to express the outcome measure, and the experimental and control groups will be statistically compared by two independent-sample *t* tests. Eight secondary outcomes will be applied to assess the efficacy of the acupuncture. Seven of these—namely, time to first defecation, abdominal pain, dosages of analgesia pump, abdominal distention, nausea, vomiting, and gastrointestinal hormones will be described usinḡχ± SD deviation and statistically compared between groups by Repeated Measures Analysis of Variance (RMANOVA). The statistical analysis of time to first defecation will be identical to that of time to first flatus. However, before performing RMANOVA, a sphericity test will be performed to determine whether the data are suitable for analysis by RMANOVA. If the data do not conform to the conditions of sphericity condition, the degree of freedom will be corrected using factor ε before the RMANOVA is performed. The categorical measure of psychological status will be analyzed using either the rank-sum test or Spearman's rank correlation.
4. Safety analysis: In accordance with the definition of adverse events, specific adverse events, their severity level, causes, and explanations will be listed. The number of adverse events, and the incidence of adverse event will be statistically described. If inter group comparison is required, the χ^2^ test or Fisher's exact test will be performed.
5. Methods of statistical analysis: An independent statistician analyzed the outcome data in accordance with the principle of intention-to-treat (ITT), that is, all subjects recruited in the group will be covered. The analysis of efficacy and safety indicators will be conducted following the principle of per-protocol (PP), that is, all subjects who completed the whole trial process without major protocol violations will be included. All statistical analysis complied with ITT population, and the results of ITT analysis will be compared with PP analysis to assess the sensitivity. All missing data will be analyzed using the last observation carried forward (LOCF) interpolation method. The qualitative data will be tested for normality, according to which independent *t* test or Mann–Whitney *U* test will be performed for groups comparison. Qualitative variables will be presented by the statistical description of χ ± SD of the normal distribution data, whereas qualitative variables will be recorded as the number of cases in each category. If the quantitative variables constitute repeated measures data, RMANOVA will be adopted for comparison, and the sphericity test will be performed to ascertain the applicability of, as well as to correct the data. The categorical variables will be represented by numbers (percentages) by χ^2^ test or Fisher's exact test *P* < .05 will be considered statistically significant on both sides in all the statistical tests. SPSS 25.0 software (SPSS, SPSS Inc, Chicago, IL) will be adopted for data statistical analysis.

## 3 Discussion

The primary factors affecting the postoperative rehabilitation of patients included gastrointestinal dysfunction, pain and inconvenience occurred after operation.^[36]^ The application of opioid analgesics can also increase the incidence of PGD; in 2009, the incidence of PGD resulting from opioid analgesics in the United States was as high as 81%.^[37]^ The prevalence of PGD in China is difficult to estimate for the lack of reliable evidence-based medicine reports. According to clinical observation, most abdominal surgery patients depressed by a series of discomfort syndrome caused by PGD, thereby seriously affecting the prognosis of patients.^[38]^ However, the usual treatment measures cannot effectively meet the requirements of patients, so clinicians and researchers must find some better measures to promote the early recovery of gastrointestinal function. The ideal postoperative rehabilitation measures can effectively promote the gastrointestinal function while exerting minimal side effects.

Acupuncture has been applied in China for over 3000 years. *Deqi* lays the basis of curative effect whether stimulating acupoints by hand acupuncture or electroacupuncture. *Deqi* refers to a special feeling (e.g., acid, numbness, heaviness, and distention) aroused towards acupuncture. Most acupoints have clear international general positions and codes, so the technology of acupuncture is easy to master and train. However, for the different philosophical basis between TCM and translational medicine, acupuncture is hard to become a mainstream treatment program. Fortunately, the emergence of molecular biology, neuroendocrinology, and immunology methods enabled researchers to apply modern technology to acupuncture research and reveal the mechanism of acupuncture.

Given the theoretical research of acupuncture and long-term clinical experience, this study hypothesizes that acupuncture may promote the recovery of gastrointestinal dysfunction after laparoscopic cholecystectomy under general anesthesia by changing the level of gastrointestinal hormones. A systematic review^[35]^ revealed that the time to first flatus after operation is of high clinical implication in the recovery of gastrointestinal motility.^[39]^ In most clinical trials regarding PGD, the time to first flatus is considered as the primary result, so it acts as the main outcome index here. PGD can cause emotional impact on patient, so the anxiety and depression of the subjects act as the secondary observation indexes as well.^[40]^ In the inclusion criteria of this study, subjects aged from 18 to 70 will be selected to cover as wide a range as possible. In the meantime, exclusion of participants taking gastrointestinal motility related drugs is critical for accurate clinical results. During the study, patients will be allowed to use rescue drugs when having intolerable symptoms; this strategy reflects the clinical practice in the real world and meets the moral obligation.

This study has some limitations. To be specific, the drug control group was designed, instead of the placebo sham acupuncture control group. In the previous experiment, we found that even intradermal acupuncture or transcutaneous electrical stimulation can exert physiological effects, which might be attributed by the specificity of acupoints. At present, there has been an absence of internationally recognized criterion and mature method of sham acupuncture.^[41]^ Moreover, it is difficult to blind subjects since most of Chinese patients have received acupuncture before, so the trial lacked sham acupuncture control group. In brief, this study protocol described a randomized, evaluated blinded, multi-center clinical controlled trial. The results of this study will evidence the effectiveness, safety, and feasibility of acupuncture for gastrointestinal dysfunction after laparoscopy; the aim of this study is to provide reliable reference for facilitating the clinical application of this therapy.

## Acknowledgments

We appreciate the help and efforts of all researchers participating in this trial.

## Author contributions

**Conceptualization:** Lisha Liu, Xiuli Yuan, Lei Yang.

**Data curation:** Lisha Liu, Xiuli Yuan.

**Formal analysis:** Lisha Liu, Xiuli Yuan.

**Funding acquisition:** Lisha Liu, Lei Yang.

**Investigation:** Lisha Liu, Xiuli Yuan, Lei Yang, Jingyuan Zhang.

**Methodology:** Lisha Liu, Xiuli Yuan, Lei Yang.

**Project administration:** Lisha Liu, Xiuli Yuan.

**Supervision:** Lisha Liu, Xiuli Yuan, Lei Yang.

**Validation:** Lisha Liu, Xiuli Yuan.

**Writing – original draft:** Lisha Liu, Xiuli Yuan, Guangqiang Huang, Lei Yang.

**Writing – review & editing:** Lisha Liu, Xiuli Yuan, Jian Huo, Jing Luo.

Lisha Liu orcid: 0000-0001-8965-4366.
